# A single-cell derived spheroid approach to dissect intratumoural heterogeneity in colorectal cancer: cell lines show changes in proteomes and therapeutic response to 5-FU

**DOI:** 10.1007/s00432-025-06418-0

**Published:** 2026-01-24

**Authors:** Helene Sophia Radloff, Michael Kohl, Thorben Sauer, Sonja Hartwig, Sven Geisler, Stefan Lehr, Timo Gemoll

**Affiliations:** 1 Section for Translational Surgical Oncology & Biobanking, Department of Surgery, University of Luebeck and University Hospital Schleswig-Holstein, Ratzeburger Allee 160, 23538 Luebeck, Germany; 2 Medical Systems Biology Group, Luebeck Institute Für Experimental Dermatology, University of Luebeck, Campus Luebeck, 23538 Lübeck, Germany; 3 Institute for Clinical Biochemistry and Pathobiochemistry, , German Diabetes Centre at the Heinrich-Heine-University Duesseldorf, Leibnitz Centre of Diabetes Research, 40225 Duesseldorf, Germany; 4 German Centre for Diabetes Research (DZD), 85764 Muenchen-Neuherberg, Germany; 5 Cell Analysis Core Facility, Institute for Systemic Inflammation Research, University of Luebeck, 23562 Luebeck, Germany

**Keywords:** Single-cell derived spheroids, Proteomics, Intratumoural heterogeneity, 5-fluorouracil, Colorectal cancer

## Abstract

**Purpose:**

Colorectal cancer (CRC) stands as a significant contributor to cancer-related mortality. Owing to its prognostic and therapeutic implications, intratumoural heterogeneity (ITH) presents a considerable challenge. We have developed an experimental framework integrating single-cell derived spheroids with proteomic profiling to facilitate a molecular, proteomic, and therapeutic characterization of intratumoural heterogeneity during CRC progression.

**Methods:**

Single cells from the commercially available colorectal cancer cell lines SW480 (primary colorectal adenocarcinoma) and SW620 (locoregional lymph node metastasis of the same donor) were isolated using fluorescence-activated cell sorting (FACS) and subsequently cultured forming spheroids. This platform allowed controlled interrogation of clonal diversity through proliferation and viability assays, alongside deep proteomic characterization using label-free liquid chromatography–mass spectrometry (LC-MS) with data-independent acquisition. To evaluate its utility for therapeutic testing, chemotherapy response was measured after 72 h of incubation with 5-fluorouracil (5-FU).

**Results:**

The single-cell derived spheroid system demonstrated significant heterogeneity, as evidenced by variations in morphology, growth dynamics, viability, and proteomic signatures. Protein profiling identified ITH-associated proteins (WDR5, CKB, IPO11, ATP6V1F, DCXR and PCCB) and underscored pathway variations including tumour suppressor and proto-oncogenic signalling, vascularization and metabolic regulation. Furthermore, individual spheroids exhibited differential sensitivities to 5-fluorouracil, demonstrating the platform’s capacity to resolve heterogeneous therapeutic responses.

**Conclusion:**

Our study establishes a robust and scalable method that integrates single-cell spheroids with proteomics to model and quantify ITH in CRC. By capturing clinically relevant diversity across morphology, viability, proteomic profiles and drug response, this approach provides a foundation for translating spheroid- and proteomics-based assays into personalized therapeutic testing.

**Supplementary Information:**

The online version contains supplementary material available at 10.1007/s00432-025-06418-0.

## Introduction

Colorectal cancer (CRC) is the third most frequently occurring cancer worldwide, with over 1.9 million new cases reported in 2020 (Morgan et al. 2023). CRC represents one of the most common cancer-associated causes of death and approximately 90% of CRC-related deaths can be attributed to metastatic progression (Chaffer and Weinberg 2011), as metastatic neoplasms pose significant challenges for effective disease management (Lambert et al. 2017). Extensive chemotherapy, immunotherapy, and targeted therapies only increase overall survival to a limited extent, because escape mechanisms and resistance occur in up to 90% of the metastatic CRC cases (Longley and Johnston 2005). Intratumoural heterogeneity (ITH) is crucial in tumour biology, treatment response, resistance development, and patient survival (McGranahan and Swanton 2017).

Tumours represent complex ecosystems that evolve under diverse selective pressures exerted by the tumour microenvironment (TME), therapeutic interventions, host and external influences (Dagogo-Jack and Shaw 2018; Shin et al. 2023). They vary across tumour regions and disease stages, thereby driving diversification of malignant and non-malignant cell populations (Dagogo-Jack and Shaw 2018). Intratumoural heterogeneity arises at multiple molecular levels, including the genome, epigenome, transcriptome, proteome, and therapy response, which profoundly influences tumour progression, metastasis, and treatment resistance (Lin et al. 2017; McGranahan and Swanton 2017; Molinari et al. 2018; Raynaud et al. 2018; Kapoor-Narula and Lenka 2022). The origin and tissue context of a tumour further predispose its heterogeneity (Hoadley et al. 2018), shaping clonal evolution between primary tumours and metastases as well as across patients (Junttila and de Sauvage 2013). CRCs frequently exhibit genomic and proteomic ITH, resulting in diverse morphological and phenotypic characteristics (Ciardiello et al. 2014; Cuyle and Prenen 2017), ultimately developing discernible subclones with distinct activated molecular signalling pathways (Zhang et al. 2014; Yin et al. 2015), metastasising capacity, tumour cell growth, and responses to therapy (Naxerova et al. 2014; Baretti et al. 2018). In this context, proteomic heterogeneity adds another dynamic layer, driven by alternative splicing, epigenetic regulation, and post-translational modifications (Bardhan and Liu 2013; Bisognin et al. 2014; Guo et al. 2025). Increased ITH is associated with adverse outcomes, including resistance to conventional and targeted therapies (Dagogo-Jack and Shaw 2018), shorter progression-free survival and reduced overall survival (Morris et al. 2016).

Many methods have been employed to address ITH, including those targeting specific molecules, varying in resolution from bulk to single-cell approaches. So-called ‘bulk’ techniques often measure traits of the overall cell population and consequently fail to assess ITH. In contrast, single-cell techniques offer new opportunities to capture spatial differences at the molecular level (Suvà and Tirosh 2019). Additionally, spheroids as three-dimensional (3D) cell aggregates serve as a superior in vitro surrogate of the ‘original’ tumour, more closely emulating its physiology and other characteristics compared to 2D (two-dimensional) cultures (Langhans 2018; Vasyutin et al. 2019). However, a comprehensive strategy that integrates functional assays with proteomic readouts to investigate ITH in single-cell derived spheroids remains absent.

This study aimed to systematically evaluate ITH in a primary colorectal cancer cell line (SW480) and its patient-matched lymph-node metastasis (SW620), enabling a parallel analysis of clonal growth behaviour, viability, therapeutic sensitivity, and proteomic signatures. Through the generation of ranked protein profiles from individual spheroids, molecular determinants of heterogeneity across CRC progression were identified. This new workflow provides a methodological foundation with potential translational value, supporting future clinical applications wherein spheroid based functional assays and proteomic readouts could inform personalised therapeutic strategies.

## Materials and methods

**Fig. 1:**
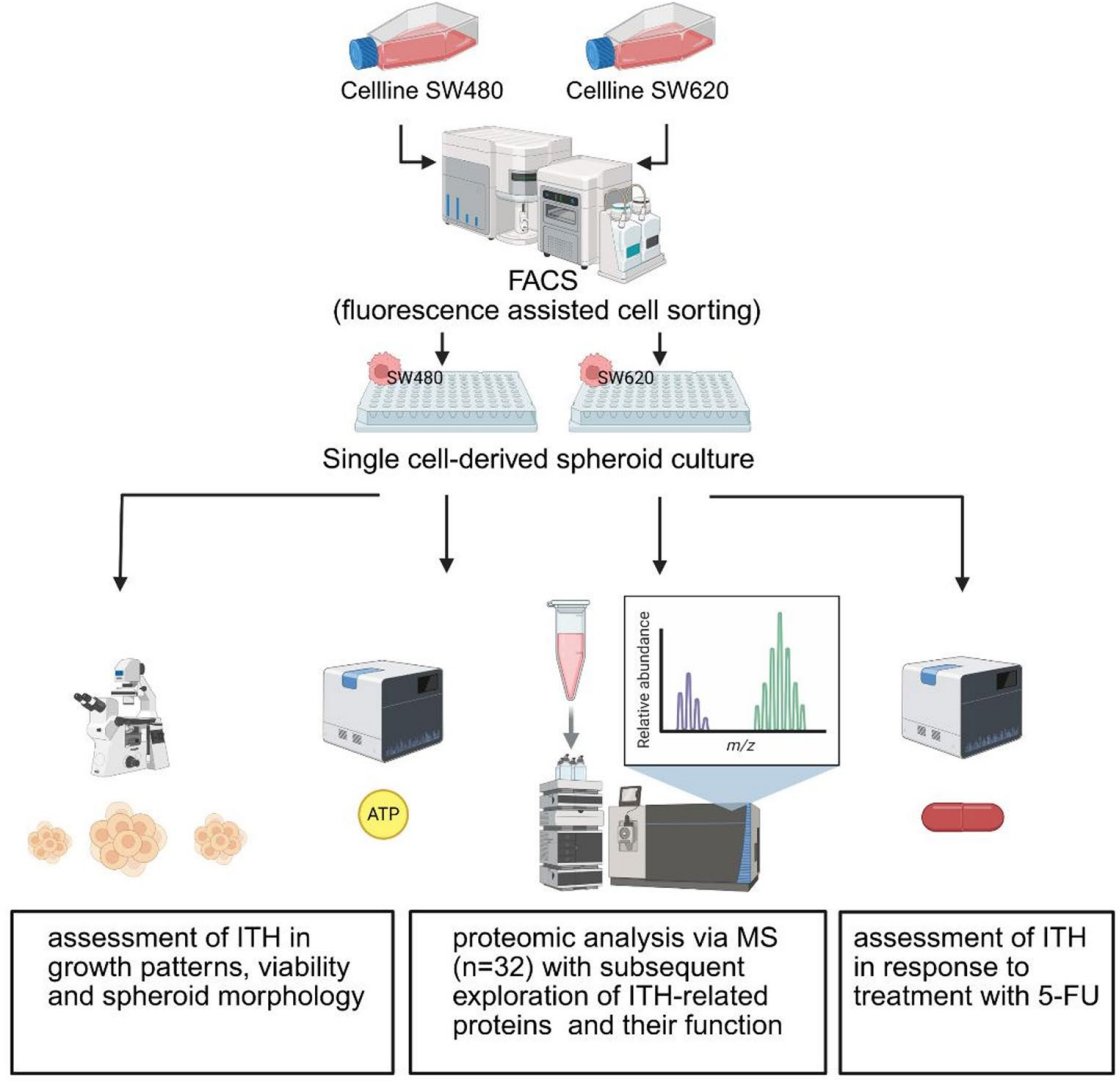
Study design. The study used a cell culture model to characterise ITH in colorectal cancer at the single-cell level. SW480 (primary adenocarcinoma) and SW620 (corresponding locoregional lymph node metastasis) cell lines were selected to investigate ITH differences in primary and metastatic tumours. Initially, cells were separated using FACS to establish single-cell-based spheroid cultures. Spheroids exhibiting morphological diversity were selected for subsequent analysis including growth behaviour, morphology, viability, and response to 5-FU treatment. Liquid chromatography-coupled mass spectrometry was used to compare the selected spheroids on the proteomic level. This figure was created using biorender

This section presents the materials and methodologies used according to the flowchart shown in Fig. 1. For all downstream characterizations, independent experimental approaches were implemented due to the requirement of spheroid lysis for viability, treatment response, and proteomic profile analyses. Size-dependent spheroid selection has been implemented in mass spectrometry (MS) analysis.

### Adherent cell culture

The commercially available CRC cell lines SW480 (RRID:CVCL_0546, ATCC® CCL-228™) and SW620 (RRID:CVCL_0547, ATCC® CCL-227™) were obtained 2008 from the American Type Culture Collection (ATCC) and preserved in a liquid nitrogen tank until usage. For cell line authentication, a short tandem repeat (STR)-analysis was performed using an aliquot derived from the corresponding cell passage used in the study, which yielded the allele combinations listed in Supplemental Table 1 (Online Resource 2). Cells were cultured at 37 °C, 5% CO_2_, and constant humidity using a Galaxy 170 R incubator (New Brunswick™, Germany). Cell growth was assessed via microscopic examination using an EVOS XL Core microscope (Invitrogen, USA). When cells reached approximately 90% confluence, they were dissociated from the culture flask using trypsin, washed with phosphate-buffered saline (PBS), and transferred to new flasks with fresh medium. Contamination with Mycoplasma was tested using the MycoScope™ MycoPlasma PCR Detection Kit (Genlantis Inc., San Diego, CA, USA).

### Single-cell-based spheroid culture after FACS sorting

To investigate the extent of ITH, we generated spheroids from single cells separately for both cell lines. Cells were sorted using Fluorescence-assisted cell sorting (FACS) with the MoFlo Legacy (Beckman Coulter Life Sciences, Indiana, USA). Initially, cells were detached from the adherent culture using trypsin and washed twice with PBS via centrifugation at 300 rpm and 20 °C for 5 min using a Rotanta 46 RS centrifuge (Hettich GmbH & Co. KG; Germany). Next, a live/dead-staining dye (Fixable Viability Stain eFluor780, Thermo Fisher, Waltham, Massachusetts, USA) was added in a 1:1500 (medium:dye) ratio and incubated in the dark for 5 min. After centrifugation, the staining solution was removed, and the pellet was resuspended in phosphate buffered saline (PBS) with 2% fetal bovine serum (FBS). Cells were sorted by forward scatter/side scatter, considering live/dead staining and cell size, ensuring only single viable cells were seeded. The gating is depicted in Supplemental Fig. 1 (Online Resource 1). Single-cell sorting was performed into a 96-well low-adherence plate, with 84 wells containing one single cell each and 12 wells containing 100 cells as control of sorting success. The culture medium was prepared using 9 ml Gibco® RPMI 1640, supplemented with 1 ml Gibco® GlutaMAX (1 X) supplement, 10 ng/ml fibroblast growth factor (FGF), 10 ng/ml of epithelial growth factor (EGF), and 200 µl of Gibco® B27 (50 X).

Differences in spheroid morphology, size, clustering density, and dissociation within the cell lines were evaluated regularly. To minimise cellular loss during harvesting, we optimised an existing in-house harvesting protocol: spheroids were localised within the well by microscopy, harvested using a Pasteur pipette, and transferred into an Eppendorf tube with minimal media. Additional samples containing culture medium only were obtained for mass spectrometric analysis to eliminate the remaining medium proteins and ensure data accuracy. Spheroid viability was assessed using the CellTiter-Glo® 3D Cell Viability Assay (Promega GmbH, Germany), following the manufacturer's instructions. Spheroid size was quantified from two-dimensional projections of microscopy images. Spheroid diameters were calculated using an Invitrogen EVOS XL core microscope in combination with the ImageJ software (1.53f, NIH Bethesda and LOCI Wisconsin, USA). For irregular or non-spherical spheroids, the maximum diameter was selected.

### Mass spectrometric measurement with the Orbitrap Exploris 480 mass spectrometer

Sixteen spheroids per cell line were selected for mass spectrometric analysis, and well labels were subsequently employed as sample identifiers. Spheroids were selected based on their diameter to ensure comprehensive representation of the entire size range (Supplemental Tables 2 and 3) (Online Resource 2). The samples were purified using the iST-Kit (In-StageTip digestion Kit, PreOmics, Planegg/Martinsried, Germany). To prevent sample dilution, the LYSE reagent was utilised at an equal volume to the samples, as instructed by the manufacturer (https://www.preomics.com/products/ist (Accessed: 18 June 2024)). Subsequently, samples were incubated with LYSE reagent in a heating block (95 °C; 1000 rpm; 10 min), sonicated (10 cycles; 30 s ON/OFF) and transferred into a cartridge to cool down to room temperature. The digestion buffer was added and incubated for 3 h (37 °C; 500 rpm). The stop buffer was added, and the samples were centrifuged at 3800 ×*g* for 1 min and washed twice using the two provided wash buffers. The cartridges were then placed into fresh collection tubes, and the provided elute buffer was used to recover peptides by centrifugation at 2800 ×*g* for 1 min in two steps and then vacuum-dried (Alpha RVC, Christ, Osterode am Harz, Germany). Lyophilised peptides were reconstituted in 1% trifluoric acid (v/v) supplemented with indexed retention time-peptides (Biognosys, Switzerland) and separated by liquid chromatography (Ultimate 3000, ThermoFisher Scientific, Massachusetts, USA). Peptides were trapped and desalted on an Acclaim PepMap C18-LC-column (ID: 75 μm; 2 cm length; ThermoFisher Scientific, Massachusetts, USA) and subsequently separated via an Aurora C18 column (25 cm × 75 μm C18 1.6 µm; IonOpticks, Middle Camberwell, Australia) using a 1 h three-step gradient at a total flow rate of 300 nl/min with buffer A (0.1% formic acid) and buffer B (80% acetonitril, 0.1% formic acid): (I) 55 min linear from 2–31% buffer B, (II) 5 min from 31–44% buffer B, (III) 1 min linear gradient increasing buffer B to 95%. MS-Data acquisition and ionisation of peptides were performed within a Nanospray Flex source (ThermoFisher Scientific, Massachusetts, USA) equipped with a column oven (PRSO-V2, Sonation lab solution, Biberach, Germany) at 40 C°, using an Orbitrap Exploris 480 mass spectrometer (OE480, ThermoFisher Scientific, Massachusetts, USA). MS data for label-free quantification was acquired in data-independent acquisition (DIA) mode. Full scan MS spectra were obtained at 120,000 resolution, m/z range of 400–1200, an automatic gain control (ACG) target value of 125%, and a maximum injection time of 50 ms. Fragmentation was performed with Higher-energy collisional dissociation (HCD) energy of 32% in 34 windows covering the range from 400–1200 (m/z) with a segment width of 24.5 (m/z), Orbitrap resolution of 30,000, AGC Target of 2000%, scan range from 200–2000 (m/z), and maximal injection time was 60 ms.

### Treatment response of individual spheroids to 5-FU therapy

**Fig. 2:**
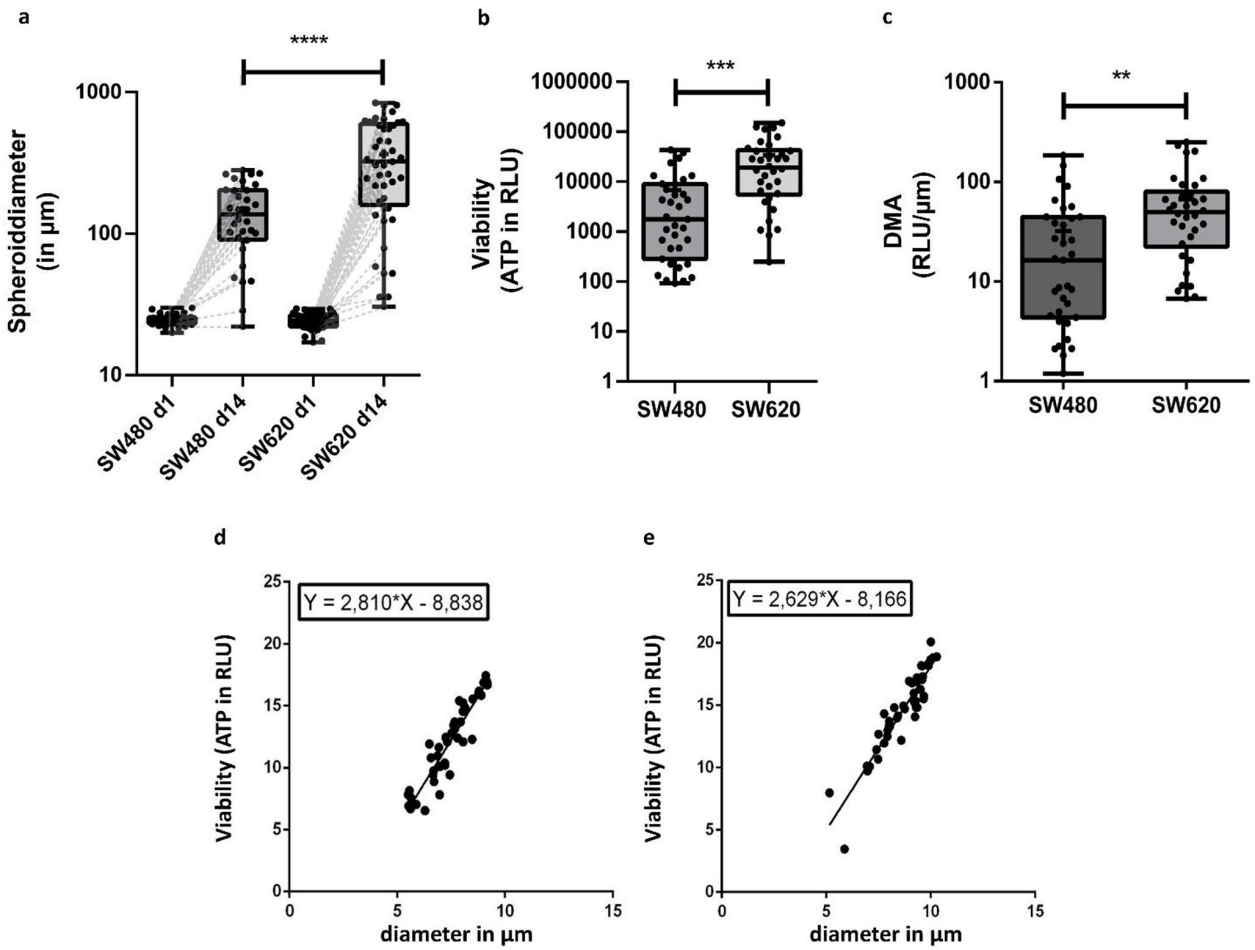
Differences in Spheroid morphology, viability and DMA within and between SW480 and SW620. The whiskers indicate the minimum and the maximum. The crossbar represents the median value and the cross shows the mean value. **(a):** Boxplots of spheroid size (given as diameter in µm) of cell lines SW480 (n = 32) and SW620 (n = 45) before and after 14 days of culture show a broad range of spheroid sizes with SW620 forming larger spheroids on average. **(b):** Boxplots of viability (ATP) for the spheroids of cell lines SW480 (n = 35) and SW620 (n = 34) after 14 days of culture reveal substantial intra-line variability and overall higher viability in SW620. The viability in relative light units (RLU) is shown on a logarithmic scale. **(c):** Boxplots of the DMA values for cell lines SW480 (n = 35) and SW620 (n = 34) are shown on a logarithmic scale. SW620 shows significantly higher DMA-values than SW480. **(a)–(c):** Asterisks indicate significance: ** p = 0.0099, *** p = 0.0003, **** p < 0.0001 (Kolmogorov Smirnov-test). **(d); (e):** Regression curves showing the positive correlation of spheroid diameter with spheroid viability of SW480 **(d)** and SW620 **(e)**. The text box indicates the regression equation. This figure was created using GraphPad Prism

Based on previously reported concentrations of 5-FU in in vitro studies (Qian et al. 2019; Källberg et al. 2023), we determined the appropriate concentration for our experimental setup by performing an IC₅₀ (half maximal inhibitory concentration) analysis across multiple concentrations (as shown in Fig. 2F and G). The final working concentration of 350 µM 5-fluorouracil was chosen based on these experimental results. To investigate the therapeutic response of single-cell spheroids, 5-fluorouracil was diluted with ultrapure water and administered to the spheroids with medium for 72 h at 37 °C. Unconditioned cell culture medium without therapeutic agents was applied to parallel control spheroids. Viability was subsequently assessed using a CellTiter Glo® viability assay. After calculating IC_50_ values, size decrease and adenosine triphosphate (ATP) amount were compared across the spheroids of the two cell lines.

### Data analysis & statistics

To account for energetic differences of the spheroids within the cell lines, we defined the ‘diameter-related metabolic activity’ (DMA) which is calculated by:

1 $$DMA = \frac{viability}{{size diameter}} $$

Spheroid characteristics (shape, clustering, budding and spheroid border) were compared between the two cell lines using Fisher’s exact test. The p-values (α = 0.05) of the differences in size, viability and DMA-values between the two cell lines were obtained using a Kolmogorov–Smirnov-test after testing for normality using a Shapiro normality-test. The relationship between size and viability within both cell lines was assessed using a regression analysis for both cell lines. Therapy response was assessed using a Wilcoxon matched-pairs signed rank test.

The mass spectrometric data were analysed as given in Supplemental Fig. 2 (Online Resource 1). The DIA mass spectra were processed using the DIA-NN v1.8 software tool (RRID:SCR_022865) (Demichev et al. 2020; Demichev et al. 2022) in library-free mode. The high-precision LC mode was used, and the RT-dependent cross-normalisation was enabled with smart profiling for library generation and single-pass mode as a neural network classifier. DIA-NN automatically determines the mass accuracy, MS1 accuracy, and scan window width. The ‘Match between runs’ option was enabled, and the human UniProtKB/Swiss-Prot database (RRID:SCR_021164) (downloaded on 06/24/2020) was utilised to generate an in silico spectral library using deep learning methods. The parameters were as follows: 1 missed cleavage was accepted, Trypsin/P was used as the protease setting, and the maximum number of variable modifications was set to 0. N-terminal methionine excision and cysteine carbamidomethylation were enabled as fixed modifications. The peptide length, precursor charge, precursor m/z range, and fragment ion m/z range were defined as 7–30, 1–4, 300–1,800, and 200–1,800, respectively. The output in 'pg_matrix' format (protein groups) was filtered at 1% false discovery rate using global q-values for protein groups and both global and run-specific q-values for the precursors.

Proteomic data were preprocessed using Perseus from the MaxQuandt software package (RRID:SCR_015753) to ensure comparability across samples. First, the data was log_2_-transformed. The raw DIA datasets were then filtered to remove proteins containing missing intensity values and exclude samples with fewer than 2500 quantified proteins to guarantee sufficient proteome depth for downstream analyses. A clustering analysis of all protein expression values was performed using a Principal Component Analysis (PCA) with the Orange Data Mining software (RRID:SCR_019811, version 3.32). To identify functional differences between protein expression clusters within SW480 we conducted a Gene Set Enrichment Analysis (GSEA) with log_2_-Fold Changes of all expression values per sample for KEGG (Kyoto Encyclopaedia of Genes and Genomes) pathways using the webtool WebGestalt: WEB-based GEne SeT AnaLysis Toolkit (RRID:SCR_006786) (Elizarraras et al. 2024).

To identify proteins associated with ITH, fold changes (FC) were calculated based on pairwise comparisons of protein expression levels among all spheroids within each cell line. For all pairwise comparisons within the respective cell line, FCs of the log_2_-transformed protein expression values were calculated and sorted according to their absolute FC values. We then selected 50 proteins with the highest absolute FC values for each comparison. The top 50 proteins per comparison (“top 50 list”) received ranking points: the highest-ranked protein received 50 points, decreasing stepwise to 1 point (lowest-ranked protein). For each protein, points were summed across all top 50 lists, and the final ranking was obtained by sorting proteins by their total points. This yielded approximately 300 proteins per cell line that accounted for most of the observed heterogeneity (“candidate ITH-driver proteins”). Heatmap visualizations were generated using row-wise Z-score normalized log₂ intensity values to illustrate relative changes in protein abundance across samples. To assess the translational relevance of our findings, we analysed publicly available single-cell RNA-sequencing data comprising 91,103 unsorted single cells from Korean and Belgian colorectal cancer patients (Lee et al. 2020) using the Single Cell Expression Atlas (George et al. 2024). This dataset includes single-cell transcriptomic profiles from multiple tumour regions and matched normal tissue. The Single Cell Expression Atlas provides standardized processing and enables investigation of gene expression patterns and cell-type-resolved localisation across studies. We qualitatively examined the spatial expression of identified candidate ITH-driving proteins (WDR5, CKB, IPO11, and ATP6V1F) across diverse cell populations (tumour core, tumour border, and adjacent non-malignant tissue) whether the protein localization appeared homogeneous, i.e., evenly distributed across the tissue section, or heterogeneous, characterized by uneven enrichment in specific regions. This classification was based on visual inspection of the relative intensity and uniformity of the signal across the analysed samples.

The single-sample Gene Set Enrichment Analysis (ssGSEA) approach provided by the Reactome software (RRID:SCR_003485, version 84) was used to compute significantly enriched Reactome pathways per cell line using potential ITH-related proteins (“candidate ITH-driver proteins”). Gene expression values were rank normalized based on absolute expression levels. For each sample, the differences were calculated between the weighted empirical cumulative distribution function (ECDF) of genes within the signature and the ECDF of all other genes in the dataset (Griss et al. 2020). The algorithm subsequently picked the 30 most differentially represented pathways in the dataset. Redundancies in pathway subgroups were removed to provide an overview of the 30 top-ranked pathways.

## Results

### Spheroid size and morphology

Following the sorting of single cells using FACS for spheroid culturing, seeded cells of both cell lines showed no significant size disparities (Fig. 2a). After 14 days of cultivation, a broad range of spheroid diameters was observed within both cell lines. The cell diameter of SW620 was 2.51 times greater than that of SW480 (p < 0.0001) and exhibited a higher coefficient of variation compared to SW480 (0.66 vs. 0.53). SW480 spheroids were more densely packed and spherical, while SW620 spheroids appeared more loosely clustered (Table 1). Illustrations of various spheroid characteristics and phenotypes are provided in the supplemental data (Supplemental Fig. 3 (Online Resource 1)).

**Table 1:** Morphological comparison of spheroids before and after 14 days of cultivation. A manual classification was performed based on morphological attributes, which include “round vs. diffuse shape”, “compact vs loosely clustered”, and “sharply bordered vs blurredly bordered margins” (reported for each cell line as spheroid counts and percentage frequency). Furthermore, the budding behaviour was evaluated, specifically whether cells detached at the edge of the spheroids. There are significant differences in the shape, clustering, and spheroid margin between the two cell lines, but no significant difference in budding behaviour was observed (Fisher’s exact test, α = 0.05)

Characteristic	Expression	SW480	SW620	*p* value
Shape	Round	15 (53.6%)	4 (8.9%)	0.0001
	Diffuse	13 (46.4%)	41 (91.1%)	
Clustering	Compact	23 (82.1%)	20 (44.4%)	0.0016
	Loose	5 (17.9%)	25 (55.6%)	
Budding	Yes	18 (64.3%)	23 (51.1%)	0.3352
Spheroid margin	Sharply bordered	19 (67.9%)	17 (37.8%)	0.0166
	Blurredly bordered	9 (32.1%)	28 (62.2%)

**Fig. 3:**
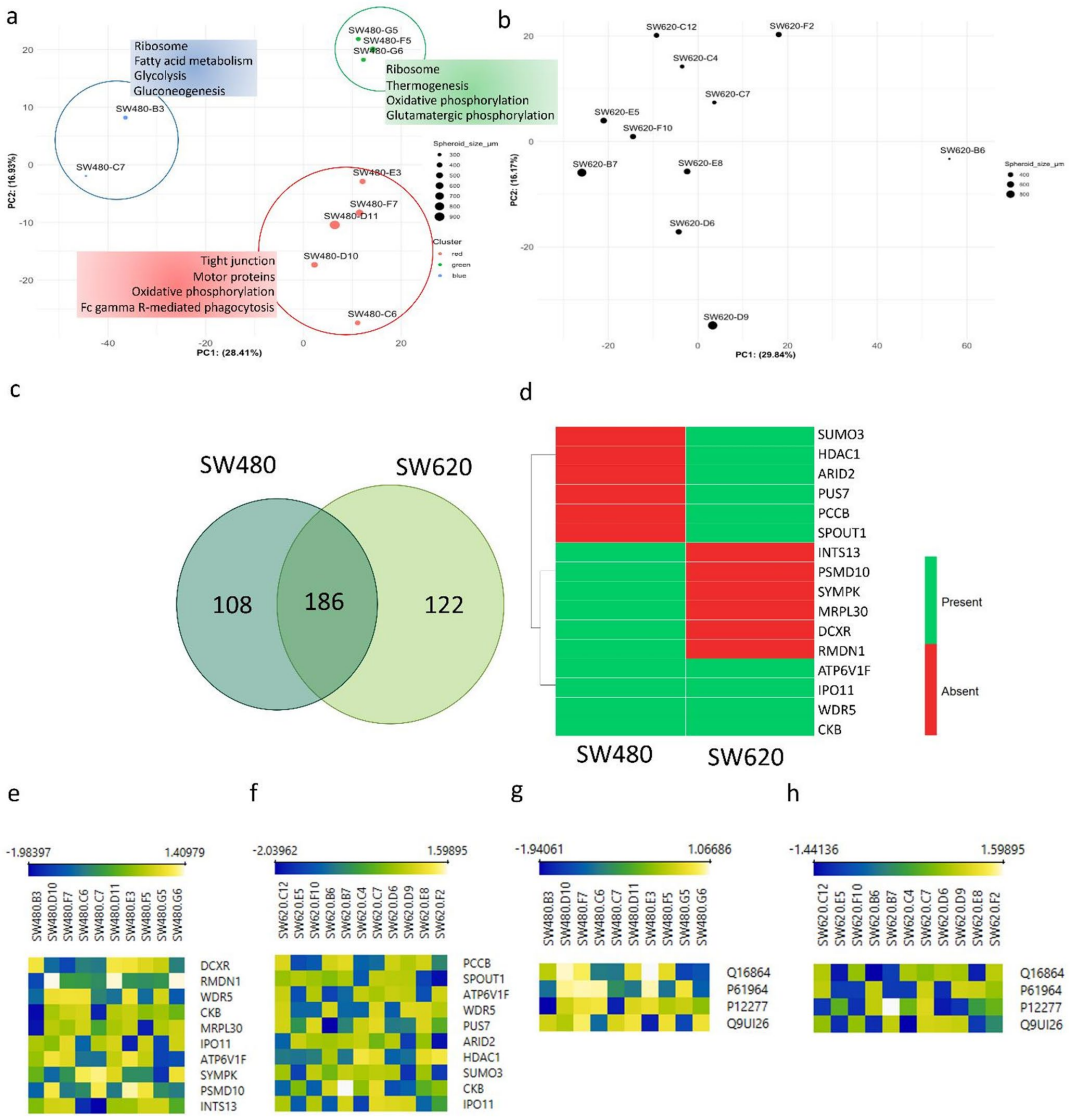
Proteomic signatures of single-cell derived spheroids. (**a**); (**b**): Principal component analysis derived from the mass spectrometry data of SW480 **(a)** and SW620 **(b)** depicting spheroid diameter as the size of the circles. SW480 spheroids exhibit three clusters indicated by different colours. The red cluster primarily comprises larger spheroids, the green cluster consists of medium-sized spheroids, and the blue cluster encompasses small spheroids. Enriched pathways per cluster are given in the text boxes. **(c):** Venn diagram showing the overlap of all ITH-driver proteins with 108 individual proteins for SW480, 122 individual proteins for SW620 and an overlap of 186 proteins between the two cell lines. **(d):** Heatmap showing the top 10 ‘candidate ITH-driver proteins’ overlap between the cell lines SW480 and SW620. The row annotations indicate the gene names and the occurrence within each cell line is shown by colour (proteins highlighted green). **(e); (f):** Heatmap of top 10 ‘candidate ITH-driver proteins’ of cell lines SW480 **(e)** and SW620 **(f**), Z-scores of protein expression (indicated by colour) reveal heterogeneous expression patterns across individual spheroids, indicating clonal proteomic diversity. The row annotation shows the gene name. Sample names of single-cell derived spheroids are displayed above. **(g), (h):** Heatmap of overlapping proteins in the heterogeneity driver-protein lists of SW480 **(g)** and SW620 **(h)**, showing variation in expression across single-cell derived spheroids. Z-scores are shown by colour scale, with gene names annotated per row. This Figure was created using Orange Data Mining software

### Viability values

ATP-based viability assays were performed to investigate the heterogeneity in spheroid viability and to examine potential differences in energy metabolism among various spheroids within the cell lines. Our results revealed that the ATP measurements in both cell lines were significantly different (*p* = 0.0003, Fig. 2b), with an average of fivefold higher viability in SW620 (SW620: 34,015 RLU (relative light units) ± 39,853 (standard deviation (SD)) than in SW480 (SW480: 6,772 RLU ± 10,900 (SD)). Regression analysis revealed a significant relationship (R^2^ > 0.8) between spheroid diameter and measured viability in both cell lines (Fig. 2d and e). The R^2^ for SW480 was 0.8716, while for SW620, it was 0.8617.

To distinguish the size-related ATP changes from effects caused by the observed heterogeneity, the diameter-related metabolic activity was calculated for both cell lines. The results showed that the metastatic cell line SW620 exhibits a significantly greater metabolic activity (SW620: 67 RLU/µm ± 64 (SD)) than the primary cell line SW480 (SW480: 32 RLU/µm ± 41 (SD), p = 0.0099, Fig. 2c).

### Proteomic profiling and pathway analysis

We used mass spectrometry to identify potential intratumoural heterogeneity on the proteomic level. The number of identified proteins correlated with spheroid size for both cell lines, with a stronger correlation for SW620 (R^2^ = 0.4941) than for SW480 (R^2^ = 0.3096) (Supplemental Fig. 4 (Online Resource 1)). After preprocessing the mass spectrometric data, the expression values of 1,655 identified proteins were used for downstream evaluations. A Principal Component Analysis revealed three clusters for SW480 closely associated with their spheroid size, while SW620 failed to exhibit any discernible clustering (Fig. 3a and b). Notably, distinct metabolic signatures correlated with spheroid size after GSEA: the cluster comprised of small spheroids exhibited significant enrichment in Glycolysis and Gluconeogenesis pathways, while clusters containing medium and large spheroids demonstrated enrichment in Oxidative Phosphorylation.

**Fig. 4:**
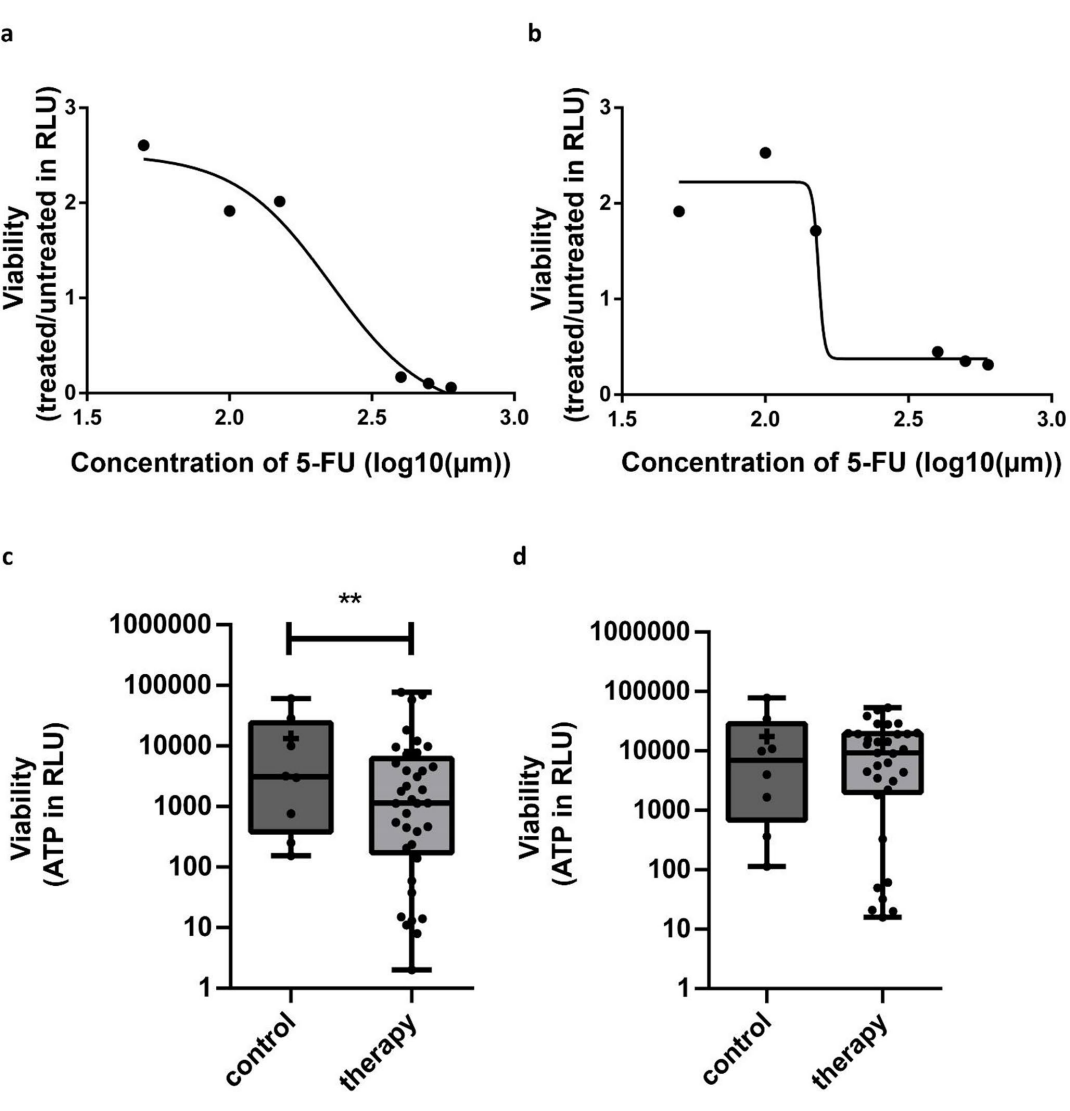
Differences in therapy response within and between SW480 and SW620. (**a**, **b**): IC_50_ curves of SW480 (**a**) and SW620 (**b**) using 5-FU as a therapeutic target. Therapy response was assessed using viability measures in RLU. Spheroids of both cell lines show resistance to treatment at low concentrations and a rapid decrease of viability at high concentrations of 5-FU. **(c, d):** Therapy response of SW480 (n = 37) (**c**) and SW620 (n = 33) (**d**) spheroids to the administration of 350 µM 5-FU in comparison to untreated samples (n = 8 for SW480 and SW620) (control). Cell line SW620 showed greater overall therapy resistance than SW480. ** *p* < 0.01 (Wilcoxon matched-pairs signed rank test). This Figure was created using GraphPad Prism

Fold changes were calculated and used for protein ranking to identify heterogeneously expressed proteins within the primary and metastatic cell lines. This resulted in two lists of 294 “candidate ITH-driver proteins” for the cell line SW480 and 308 for the cell line SW620. With an overlap of 186 proteins, 122 and 108 were identified as specific ITH drivers for SW480 and SW620, respectively (Fig. 3c and d). The complete lists are given in the supplement (Supplemental Tables 4 and 5 (Online Resource 2)).

A comparison of the top 10 ‘candidate ITH-driver proteins’ of SW480 (DCXR, RMDN1, WDR5, CKB, MRPL30, IPO11, ATP6V1F, SYMPK, PSMD10, INTS13, Fig. 3e) with those of SW620 (PCCB, SPOUT1, ATP6V1F, WDR5, PUS7, ARID2, HDAC1, SUMO3, CKB, IPO11, Fig. 3f) reveals an overlap of four proteins, namely WD repeat-containing protein 5 (WDR5), Creatine kinase B-type (CKB), Importin-11 (IPO11), and V-type proton ATPase subunit F (ATP6V1F) (Fig. 3g and h). Analysis of patient-derived single-cell RNA sequencing data confirmed heterogeneous expression of the overlapping ITH-driver proteins by demonstrating an enrichment in distinct tumour cell populations (Supplemental Fig. 5–8 (Online Resource 1)).

Following a pathway analysis of the ‘candidate ITH-driver proteins’ utilizing the ssGSEA method provided by Reactome (RRID:SCR_003485), we sought to identify any enriched pathways associated with ITH. For the SW480 cell line, 895 signalling pathways were discovered, while 1,098 pathways were identified for the SW620 cell line (Supplemental Tables 6 and 7 (Online Resource 2)). In spheroids of SW480, the implicated pathways comprised tumour suppressor genes, proto-oncogenes, and transcription factors, while SW620 spheroids showed activated pathways of vascularization and ATP-provision (Supplemental Fig. 9a and b (Online Resource 1)). Interestingly, the pathway analysis of SW620 revealed “RHO GTPase cycle” and “Creatine Metabolism” as part of the top-ranked 30 ne6tworks including the ITH-driver protein Creatine Kinase B-type (CKB).

### Response to chemotherapy with 5-FU

Therapy experiments were performed with 350 µM 5-FU after determining therapeutic concentration using an IC_50_ analysis (Fig. 4a) and are shown in Fig. 4b. For the SW480 cell line, the average viability of treated spheroids (mean: 8.2 RLU; standard deviation: 18.7) demonstrated a 38.3% reduction compared to that of untreated spheroids (mean: 13.3 RLU, p = 0.0078). For the SW620 cell line, there was a 23.2% reduction in the mean viability of the treated spheroids (mean: 13.4 RLU; SD: 14.2) compared to the mean viability of the untreated spheroids (17.5 RLU, p = 0.3828). In both cell lines, a subset of spheroids exhibited resistance to treatment with 5-FU as illustrated in Fig. 4b and detailed in Supplemental Table 8 (Online Resource 2). To assess the impact of morphology on treatment response, spheroid diameter, post-treatment viability, and morphological characteristics were analysed (Supplemental Table 8 (Online Resource 2)). A size-dependent correlation to therapy resistance was evident in both cell lines, with a more pronounced spheroid-size dependence of therapy resistance in SW480 (R^2^ = 0.4809) than in SW620 (R^2^ = 0.2047) (Supplemental Fig. 1(Online Resource 1)).

## Discussion

This study aimed to establish an integrated single-cell spheroid and proteomics workflow to model intratumoural heterogeneity (ITH) in colorectal cancer. Using this platform, we linked clonal variability to distinct proteomic signatures as well as differences in morphology, growth behaviour, viability, and chemotherapy response. By advancing single-cell derived spheroid cultivation of the paired CRC cell lines SW480 and SW620, we provide new insights into molecular and phenotypic divergence associated with tumour progression and metastasis. To our knowledge, a comprehensive mass spectrometry-based characterization of ITH in CRC cell lines has not yet been reported. The protocol developed here highlights the value of cell-line-based spheroid systems as controllable, scalable models for interrogating heterogeneity, particularly at the proteomic level. Importantly, our findings show that proteomic variability at the spheroid level is functionally meaningful, reflecting clonal differences that may contribute to tumour aggressiveness, metastatic potential, and therapeutic resistance (Joung et al. 2017; Oh et al. 2019; Fu et al. 2025).

### Single-cell derived spheroids show different growth rates, morphological heterogeneity, and heterogeneous intracellular ATP levels

This study found that the selected cells from both cell lines formed spheroids of varying sizes after a 14-day culture period. A possible explanation for the vast size ranges within the cell line SW620 might be the malignant potential of metastasised cells, including different cell growth rates. Although the spheroid size was estimated from 2D images, this finding is consistent with those of Tomita et al., who observed spheroids growing compactly (E-type) and loosely clustered (R-type) in both cell lines, including an increased malignant potential for R-type cells (Tomita et al. 1992). In line, we detected a significantly higher proportion of R-type cells in SW620 (55.6%) than in SW480 cells (17.9%, *p* = 0.0016, Table 1), validating morphological and size differences between the primary and the metastatic successors.

Following the question of whether single-cell spheroids from SW480 and SW620 show heterogeneous viability properties, this study detected a distinct pattern of ATP values in both cell lines with a strong relationship between spheroid diameter and viability after culturing SW480 and SW620 single-cell derived spheroids. The positive correlation between spheroid diameter and ATP levels observed in our study likely reflects, in part, the higher number of cells in larger spheroids. However, ATP content within spheroids is influenced not only by cell number, but also by metabolic adaptations, nutrient and oxygen gradients as well as by the development of necrotic cores in larger spheroids (Weiswald et al. 2015). Additionally, loosely clustered spheroids may exhibit reduced hypoxia and necrosis compared to more compact spheroids. Therefore, observed differences may be attributable to a combination of biological and methodological factors, rather than solely reflecting intrinsic metabolic activity.

Against this background, our defined 'diameter-related metabolic activity' (DMA) demonstrated heterogeneity across the analysed cell lines and indicated the existence of metabolically distinct subpopulations (Fig. 2c**)**. According to Fiorillo et al., ATP-rich tumour cells are associated with tumour aggressiveness, multidrug resistance, and spontaneous metastasis. It was hypothesised that these energetically adapted tumour cell populations would likely better resist external selection pressures giving them a survival advantage and favouring recurrence and metastasis (Fiorillo et al. 2021).

### Heterogeneous expression changes are detectable at the proteome level

To ensure the reliable identification of differentially expressed proteins, a stringent filtering strategy was employed. While this approach may underestimate the full extent of proteomic heterogeneity, our proteomics approach detected heterogeneously expressed proteins within single-cell derived spheroids of SW480 (primary tumour) and its paired metastatic counterpart SW620. Protein expression values demonstrated size-dependent clustering in SW480 cells. However, SW620 cells did not exhibit significant clustering, suggesting increased proteomic heterogeneity within this cell line (Fig. 3a and b).

The analysis of ITH-associated proteins showed an overlap of 186 proteins in both cell lines. 108 proteins are identified as potential ITH drivers unique to the cell line SW480 and 122 for SW620 (Fig. 3c and d). Regarding the overlap, four ITH-related proteins are shared between SW480 and SW620, ranking within the top ten of each cell line, and might significantly impact ITH in general. Interestingly, all proteins play essential roles during cancer progression and demonstrated enrichments in distinct tumour cell populations (Supplemental Fig. 5–8 (Online Resource** 1)**): WDR5, CKB, IPO11, and ATP6V1F. The WD repeat-containing protein 5 (WDR5) is involved in the pathways of chromatin organization, which plays a role in carcinogenesis and cancer therapy response (Zhou et al. 2021). Aberrant expression of WDR5 is associated with several cancers, including bladder cancer, leukaemia, and prostate cancer and has been shown to facilitate cell growth, metastasis, and chemoresistance (Krivtsov and Armstrong 2007; Chen et al. 2015; Gu et al. 2017). Creatine Kinase B-type (CKB) reversibly catalyses the phosphorylation of creatine phosphate, producing creatine and ATP (Lin et al. 1994). This enzyme is also commonly expressed in CRCs, premalignant polyps, and CRC metastases (Balasubramani et al. 2006). It is also part of the pathways “RHO GTPase cycle” and “Creatine Metabolism”, which was detected as a significant pathway of the ‘candidate ITH-driver proteins’ (Supplemental Fig. 9a and b (Online Resource 1)). Importin-11 (IPO11) is a nuclear transport receptor for protein import and simultaneously serves as a proto-oncogene and a tumour suppressor gene. IPO11 was identified to be important for β-Catenin-mediated transcription in APC mutant CRC cells and found to be elevated in CRCs (Mis et al. 2019). Lastly, V-type proton ATPase subunit F (ATP6V1F) is essential for ATP hydrolysis and proton translocation, maintaining intracellular pH and acidifying the extracellular environment, thus playing an important role in energy metabolism (Marshansky et al. 2014).

Furthermore, we identified proteins specific to the ITH in SW480 and SW620, respectively. As the top heterogeneity driver protein for the SW480 cell line, L-xylulose reductase (DCXR) was identified. This enzyme is involved in the NADPH-dependent reduction of various sugars and the uronate cycle of glucose metabolism (Ebert et al. 2015). It is an important downstream effector in one of the detected ITH-pathways “Formation of Xylulose 5-Phosphat” (Supplemental Fig. 9a and b (Online Resource 1)), which serves the digestion of intermediates of the pentosephosphate metabolism. For SW620, we detected the mitochondrial propionyl-CoA carboxylase β-chain (PCCB) as the top-ranked driver for ITH. PCCB is involved in the catabolism of metabolites such as odd-chain fatty acids and the amino acids isoleucine, methionine, valine, and threonine (Kalousek et al. 1980; Jiang et al. 2005). This protein is also involved in the pathway “Metabolism of vitamins and cofactors”, which we already described as one of the significantly enriched pathways of the candidate ITH-driver proteins (Supplemental Fig. 9a and b (Online Resource 1)). The identified proteins could potentially serve as candidate therapeutic targets for further validation.

Gene Set Enrichment Analysis of KEGG pathways was performed to investigate functional differences among SW480 spheroid clusters. Interestingly, large and medium spheroids showed enrichment in oxidative phosphorylation, a pathway known to be important in tumour progression, invasion and metastasis (Tufail et al. 2024). Tumour cells frequently enhance glycolytic metabolism to support growth and also modulate their metabolic response to environmental fluctuations or external stimuli (Zhao et al. 2023; Tufail et al. 2024). The enrichment of oxidative phosphorylation in medium and larger spheroids compared to smaller spheroids underlines the relationship of spheroid size, ATP-amount and tumour aggressiveness described above.

### ITH within the response to 5-FU therapy

Our results from the 5-FU treatment experiment confirm the hypothesis that ITH is an important influencing factor for therapeutic resistance. Consistent with our findings, Arul et al. showed that colorectal cancer cell lines exhibit heterogeneous responses to 5-FU, with SW620 in particular demonstrating substantial resistance (Arul et al. 2017). In SW480 spheroids, increased compactness and size correlated with enhanced resistance, potentially attributable to diminished drug penetration or metabolic adaptations as previously discussed. Conversely, the less dense SW620 spheroids exhibited a stronger overall therapy resistance with a weaker size-dependence, indicative of a greater degree of heterogeneity (Supplemental Fig. 10 (Online Resource 1)). These findings suggest that both spheroid morphology and metabolic adaptation are contributing factors to therapeutic tolerance. This aligns with the observations presented by Luo et al., indicating that heterogeneity in the expression of proteins is linked to drug resistance between primary CRCs and lymph node metastases (Luo et al. 2016). Additionally, the authors showed an increased invasion and migration ability in the SW620 cell line, which could be aligned with the results of our study concerning activated invasion and migration pathways in SW620 (Supplemental Fig. 9b (Online Resource 1)). Interestingly, Zhou et al. and Pitroda et al. (Pitroda et al. 2009; Zhou et al. 2012) have previously demonstrated a relationship between intracellular ATP levels, a glycolytic tumour phenotype and heightened resistance to chemotherapy. This follows our earlier observations and the high ATP levels we detected in SW620 spheroids. As hormetic or adaptive response phenomena at lower chemotherapeutic concentrations (Fig. 4a) might influence chemotherapeutic response, further research might explore live/dead flow cytometry or microscopy (Calabrese 2008; Udelnow et al. 2011). The main limitation to the therapeutic assay is that spheroids lack the full microenvironment, immune components, and stromal interactions present in patient tissue. Furthermore, the viability assay needs spheroid lysis, which renders longitudinal assessment of the same spheroid before and after therapy impossible. Consequently, while our results provide mechanistic insights and identify potential biomarkers, direct quantitative translation to patient tumours should be interpreted cautiously.

## Conclusion

In conclusion, this study introduces an integrated single-cell spheroid and proteomics workflow that effectively simulates intratumoural heterogeneity in colorectal cancer encompassing growth, morphology, viability, protein expression, and treatment response. This approach provides the first mass-spectrometry-based characterization of clonal diversity in paired CRC cell lines, thereby identifying key molecular determinants of heterogeneous drug sensitivity. The methodology offers a robust basis for clinically oriented applications, wherein spheroid-based functional testing coupled with proteomic profiling may support personalised therapy selection and prediction of treatment response. Future work expanding this platform to additional CRC models and patient derived organoids will further strengthen its translational potential.

## Supplementary Information

Below is the link to the electronic supplementary material.

Supplementary Material 1

Supplementary Material 2

## Data Availability

The datasets supporting the conclusions of this article are included within the article and its additional files. The mass spectrometry proteomics data have been deposited at ProteomeX-change Consortium (Deutsch *et al.*, 2017) via the PRIDE (Perez-Riverol *et al.*, 2019) partner repository within the data set identifier PXD053393 and are available from the corresponding author on reasonable request.

## Abbreviations

ACG: Automatic gain control
ATCC: American type culture collection
ATP: Adenosine triphosphate
CRC: Colorectal cancer
2D/ 3D: Two-dimensional/ three-dimensional
DIA: Data independent acquisition
DMA: Diameter-related metabolic activity
ECDF: Empirical cumulative distribution function
EGF: Endothelial growth factor
FACS: Fluorescence-activated cell sorting
FC: Fold change
FGF: Fibroblast growth factor
5-FU: 5-Fluorouracil
(ss)GSEA: (Single-sample) Gene set enrichment analysis
KEGG: Kyoto encyclopaedia of genes and genomes
HCD: Higher-energy collisional dissociation
IC_50_: Half maximal inhibitory concentration
ITH: Intratumoural heterogeneity
LC-MS: Liquid chromatography coupled mass spectrometry
PBS: Phosphate buffered saline
PCA: Principal component analysis
RLU: Relative light units
SD: Standard deviation
STR: Short tandem repeats
TME: Tumour microenvironment
