# Supplementary material for: A single-cell derived spheroid approach to dissect intratumoural heterogeneity in colorectal cancer: cell lines show changes in proteomes and therapeutic response to 5-FU
Source: J Cancer Res Clin Oncol. 2026 Jan 24;152(1):43. doi: 10.1007/s00432-025-06418-0 (PMC12831780; doi:10.1007/s00432-025-06418-0)
Supplement: Supplementary file 1 — Supplementary Material 1 [file 432_2025_6418_MOESM1_ESM.docx]

Supplementary Figures: Journal of Cancer Research and Clinical Oncology

A Single-Cell Derived Spheroid Approach to Dissect Intratumoral Heterogeneity in Colorectal Cancer: Cell lines Show Changes in Proteomes and Therapeutic Response to 5-FU.

**Helene Sophia Radloff¹, Michael Kohl¹^,2^, Thorben Sauer¹, Sonja Hartwig^3,4,^, Sven Geisler^5^, Stefan Lehr^3,4^ and Timo Gemoll*¹**

***Correspondence:**

Prof. Timo Gemoll, Ph.D.

Section for Translational Surgical Oncology & Biobanking,

Department of Surgery

University of Lübeck and University Hospital Schleswig-Holstein, Campus Lübeck

Ratzeburger Allee 160

D-23538 Lübeck, Germany

Phone: +49-451 3101 8703

Telefax: +49-451 3101 8704

E-mail: [Timo.Gemoll@uni-luebeck.de](mailto:Timo.Gemoll@uni-luebeck.de)

# Supplementary Figures

**
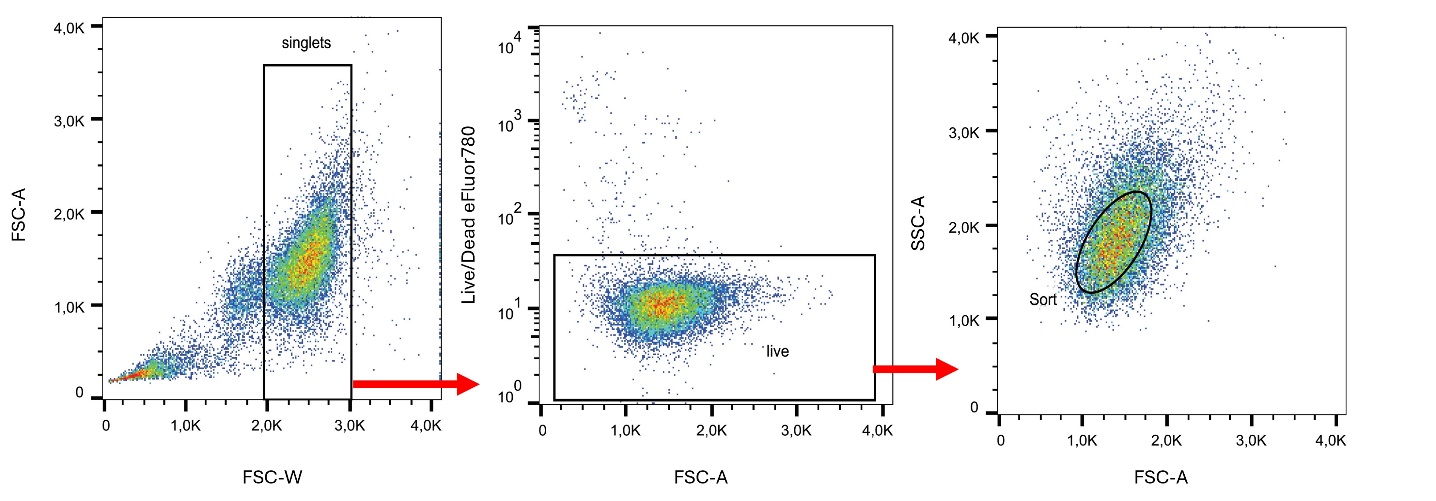
**

**Supplemental Figure 1 Gating for FACS sorting of single cells in FlowJo Analysis Software.** Singlets = Gate Singlets, only single cells are picked for sorting, Live = Gate Live, only viable cells are picked for sorting, Sort = Gate only living single cells. Gate Sort = cells with a size of 1000 – 2000 relative units (FSC-A) and a granularity of 1200 – 2400 (SSC-A) are sorted, really big cells (FSC-A 2000 – 4000) are not picked for sorting, heavily granulated cells (SSC-A > 2500) are not picked for sorting.

**
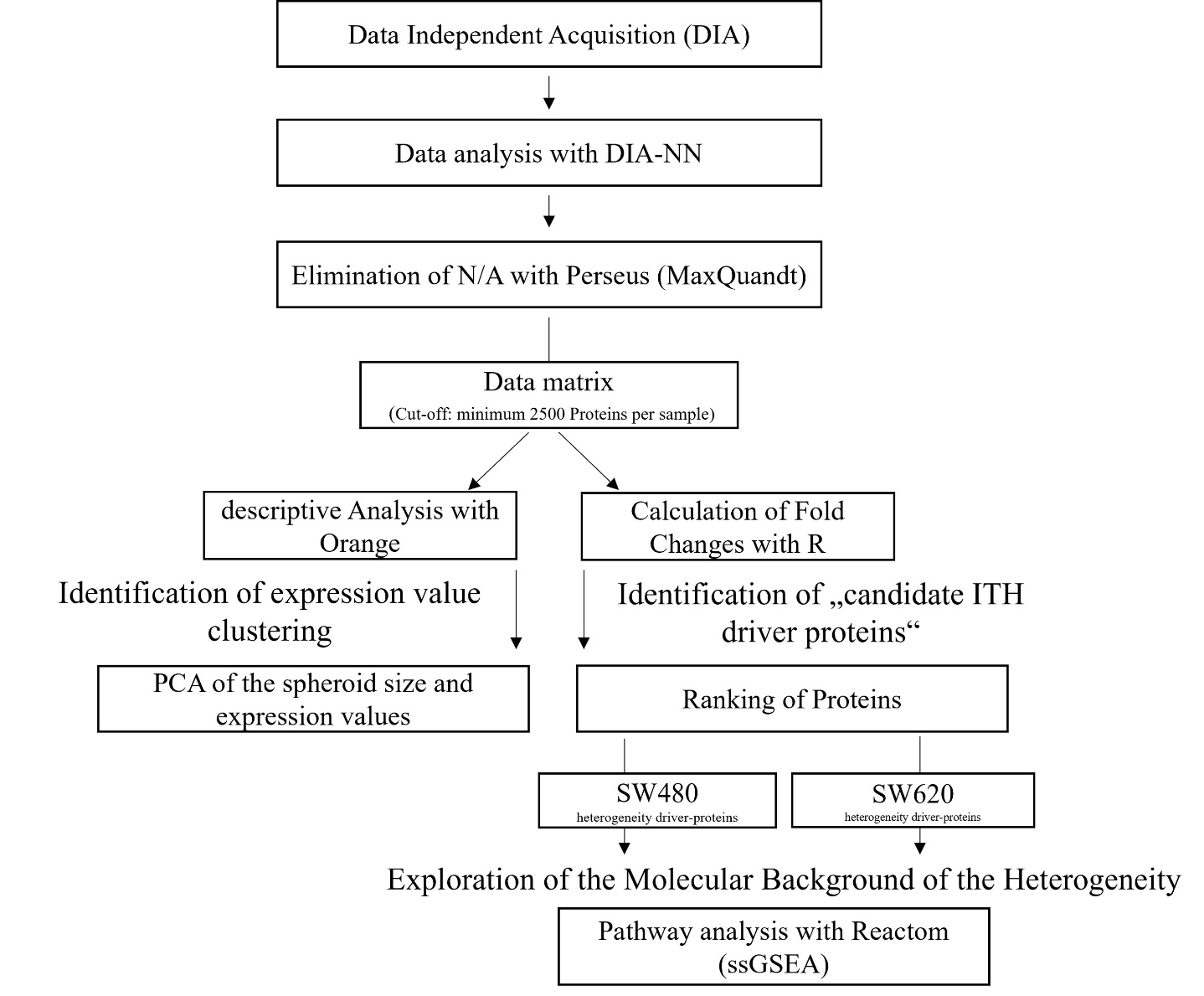
**

**Supplemental Figure 2 Design of the data evaluation.** MS data was acquired in the DIA mode of the MS device and pre-processed with the DIA-NN v1.8 software (RRID:SCR_022865). Subsequently, missing values were eliminated using the Perseus software. Orange was used for clustering analysis.  FC values for each protein were computed from all possible pairwise sample combinations to calculate a rank list of the proteins with the highest impact on the ITH separately in both SW480 and SW620. Significantly enriched Reactome pathways were calculated from these lists of ‘candidate ITH-driver proteins’ using the ssGSEA tool provided by Reactome (RRID:SCR_003485, version 84).

**
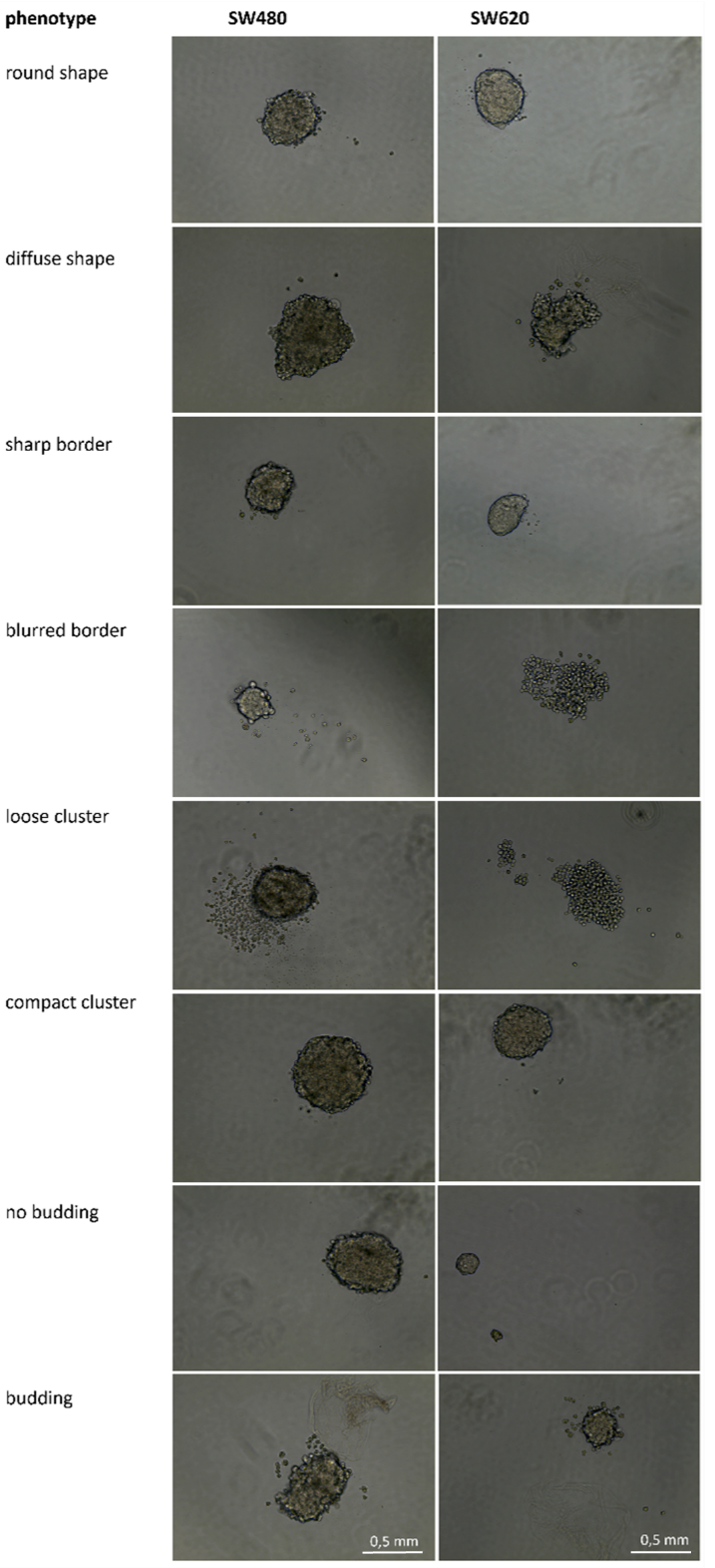
**

**Supplemental Figure 3 Spheroid morphology of SW480 and SW620 spheroids after single cell-derived spheroid culture.** Different morphologies emerged after the cell culture. They are described as either round or diffusely shaped, compactly or loosely clustered, with a sharp or blurry boundary and with or without budding. There are examples given for the different phenotypical descriptions.


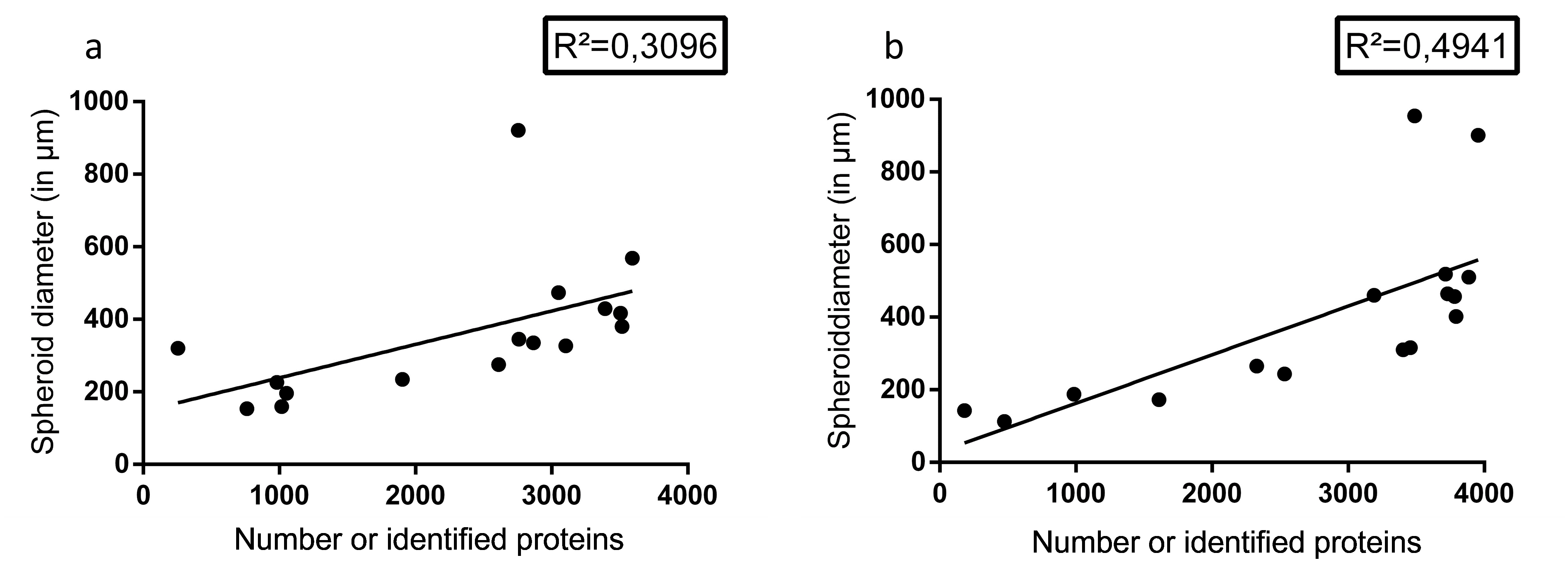


**Supplemental Figure 4** **Number of protein IDs per spheroid of SW480 (a) and SW620 (b) depending on spheroid size.** The x-axis shows the number of identified proteins per spheroid while the y-axis shows the spheroid diameter.


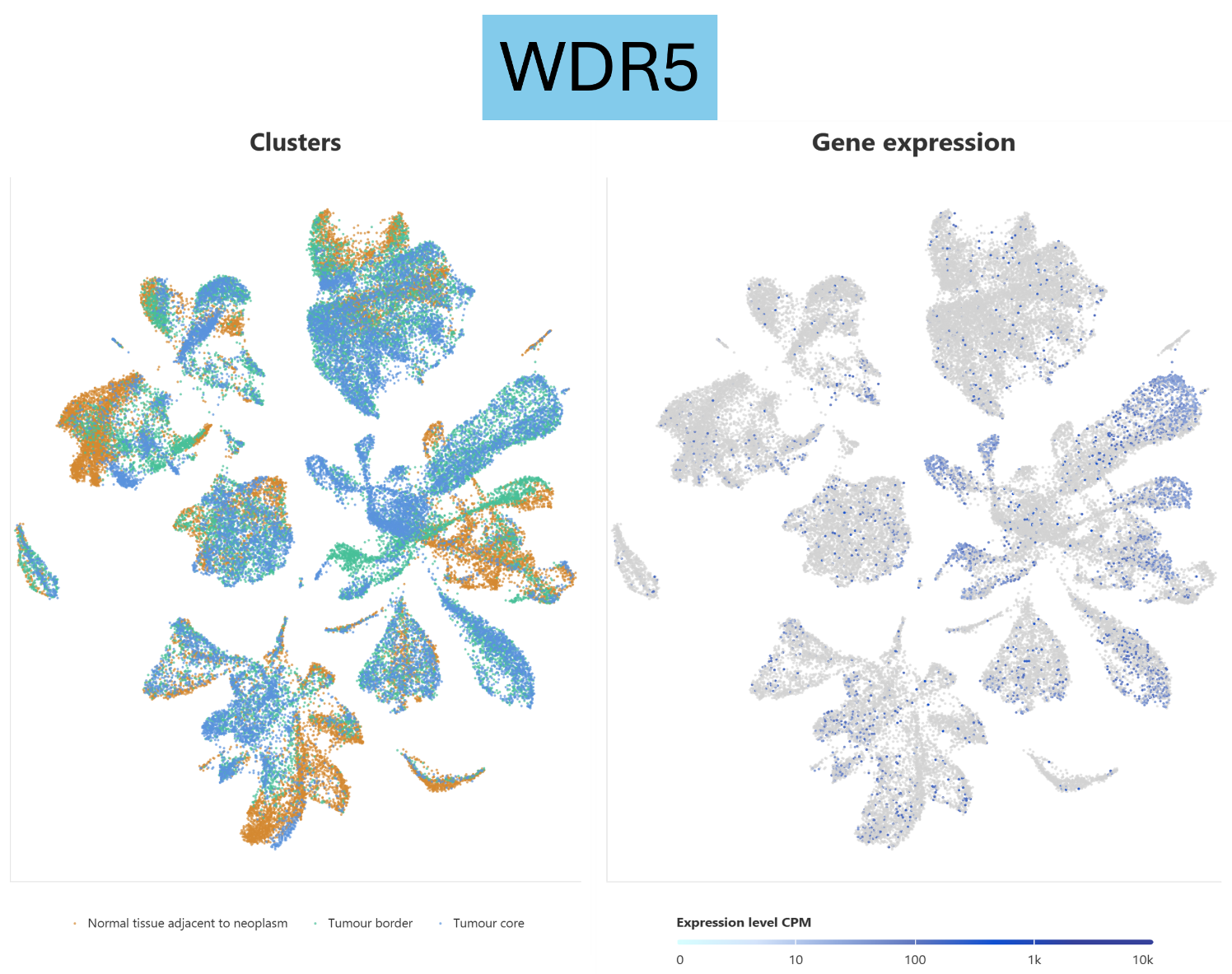
**Supplemental Figure 5 Spatial heterogeneity of gene expression for the overlapping heterogeneity (ITH)–driver protein WDR5.** Publicly available single-cell RNA-sequencing data from colorectal cancer patients (1) were used to validate the clinical relevance of candidate ITH-driving proteins identified in our cell line–based analyses (WDR5, CKB, IPO11, ATP6V1F). The UMAP on the left shows the distribution of cell populations derived from the tumour core (blue), tumour border (green), and adjacent non-malignant tissue (orange). The right UMAP illustrates the spatial expression patterns of the selected proteins. We qualitatively assessed whether expression appeared homogeneous (evenly distributed across tissue regions) or heterogeneous (enriched in specific compartments) based on visual inspection of relative signal intensity and uniformity.


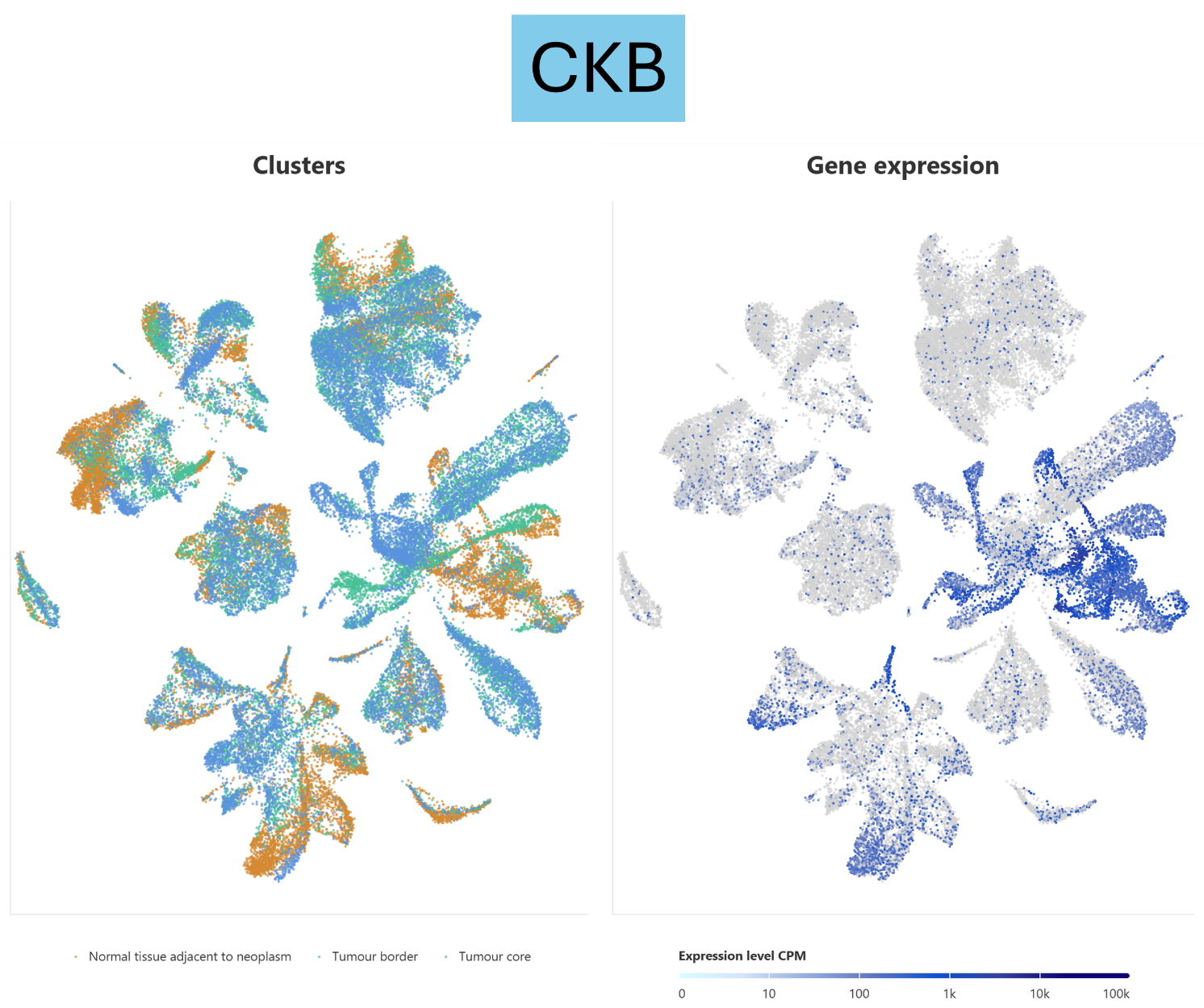


**Supplemental Figure 6 Spatial heterogeneity of gene expression for the overlapping heterogeneity (ITH)–driver protein CKB.** Publicly available single-cell RNA-sequencing data from colorectal cancer patients (1) were used to validate the clinical relevance of candidate ITH-driving proteins identified in our cell line–based analyses (WDR5, CKB, IPO11, ATP6V1F). The UMAP on the left shows the distribution of cell populations derived from the tumour core (blue), tumour border (green), and adjacent non-malignant tissue (orange). The right UMAP illustrates the spatial expression patterns of the selected proteins. We qualitatively assessed whether expression appeared homogeneous (evenly distributed across tissue regions) or heterogeneous (enriched in specific compartments) based on visual inspection of relative signal intensity and uniformity.


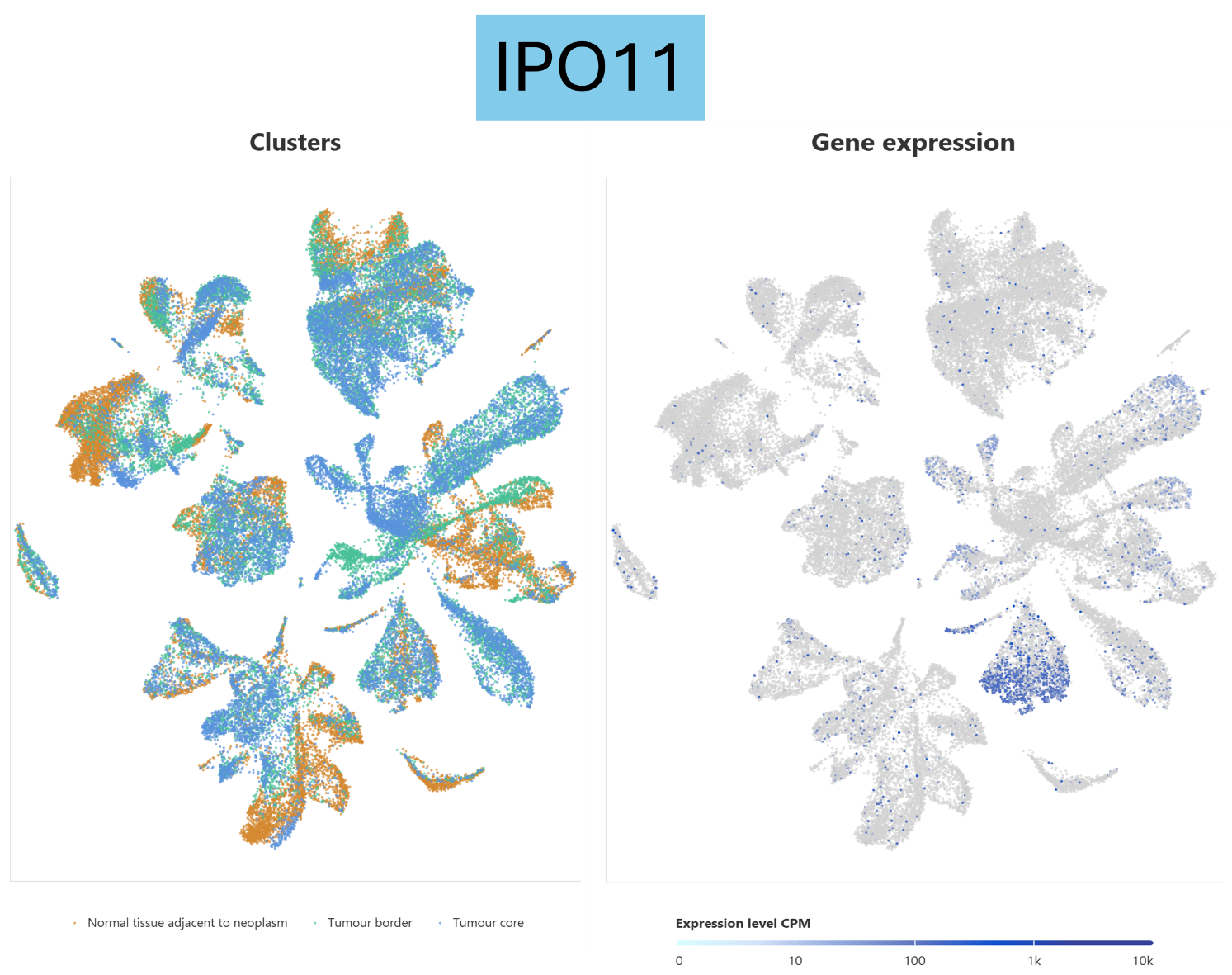


**Supplemental Figure 7 Spatial heterogeneity of gene expression for the overlapping heterogeneity (ITH)–driver protein IPO11.** Publicly available single-cell RNA-sequencing data from colorectal cancer patients (1) were used to validate the clinical relevance of candidate ITH-driving proteins identified in our cell line–based analyses (WDR5, CKB, IPO11, ATP6V1F). The UMAP on the left shows the distribution of cell populations derived from the tumour core (blue), tumour border (green), and adjacent non-malignant tissue (orange). The right UMAP illustrates the spatial expression patterns of the selected proteins. We qualitatively assessed whether expression appeared homogeneous (evenly distributed across tissue regions) or heterogeneous (enriched in specific compartments) based on visual inspection of relative signal intensity and uniformity.


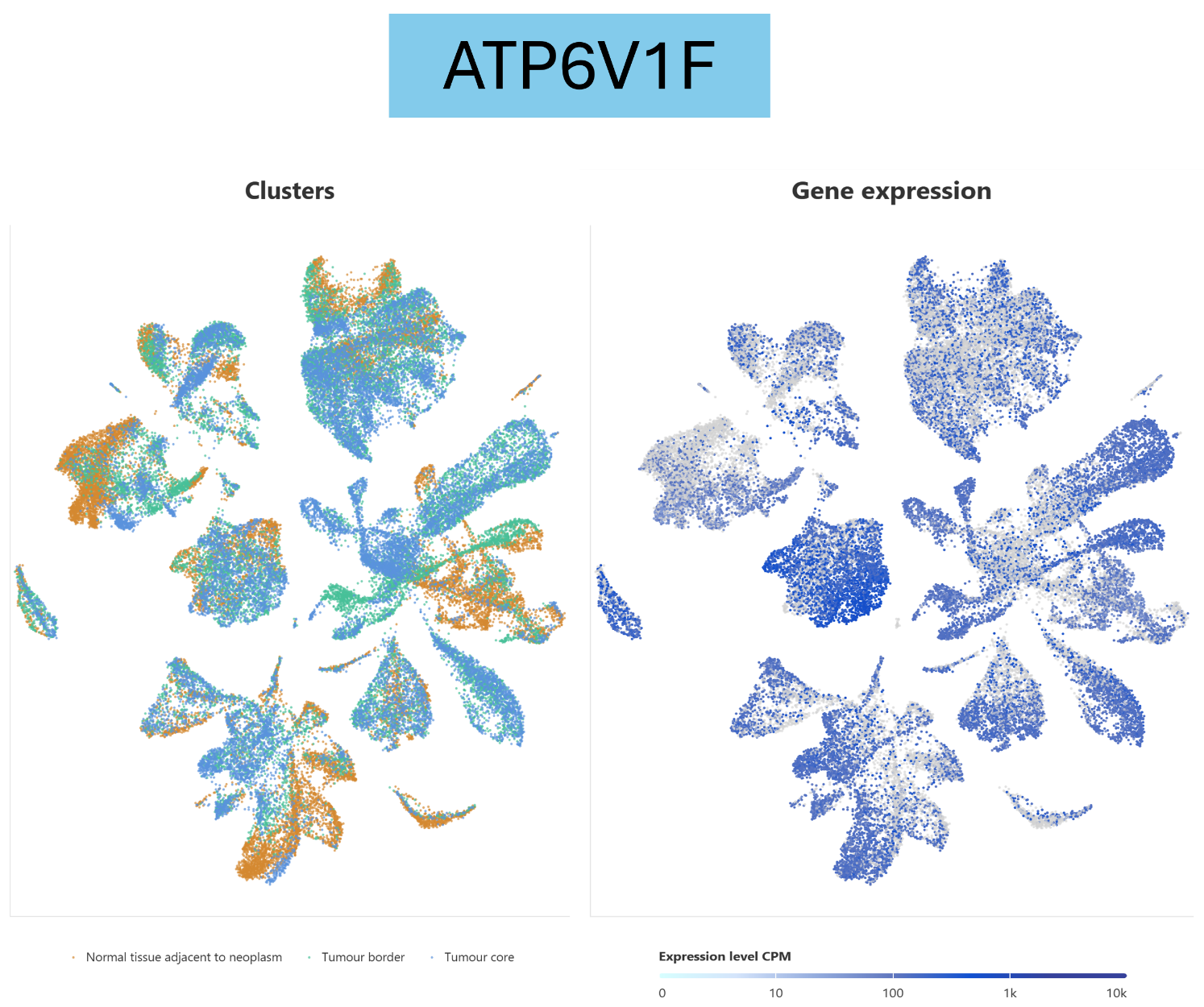


**Supplemental Figure 8 Spatial heterogeneity of gene expression for the overlapping heterogeneity (ITH)–driver protein ATP6V1F.** Publicly available single-cell RNA-sequencing data from colorectal cancer patients (1) were used to validate the clinical relevance of candidate ITH-driving proteins identified in our cell line–based analyses (WDR5, CKB, IPO11, ATP6V1F). The UMAP on the left shows the distribution of cell populations derived from the tumour core (blue), tumour border (green), and adjacent non-malignant tissue (orange). The right UMAP illustrates the spatial expression patterns of the selected proteins. We qualitatively assessed whether expression appeared homogeneous (evenly distributed across tissue regions) or heterogeneous (enriched in specific compartments) based on visual inspection of relative signal intensity and uniformity.


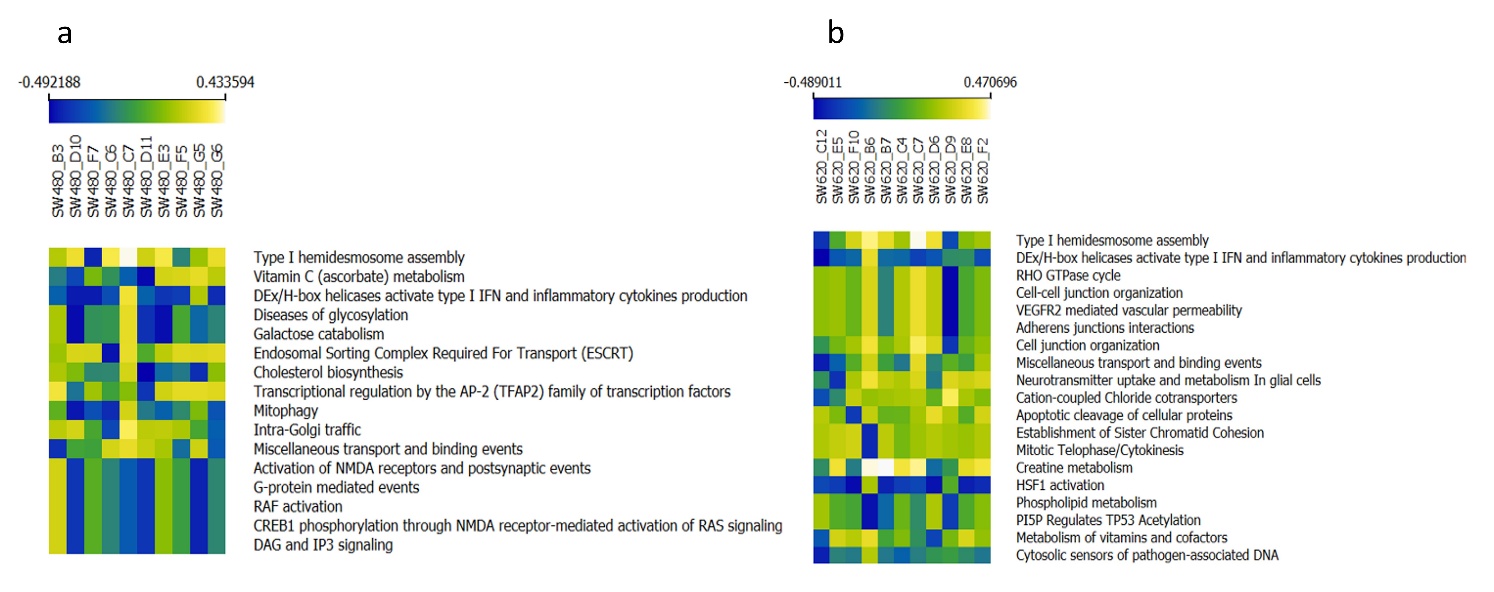


**Supplemental Figure 9** Heatmap of the Reactome/ssGSEA results for SW480 **(a)** and SW620 **(b)**. The x-axis shows the identifiers of all included samples. The y-axis depicts the most significantly enriched signalling pathways for SW480 and SW620. The colours show the enrichment score given by ssGSEA.


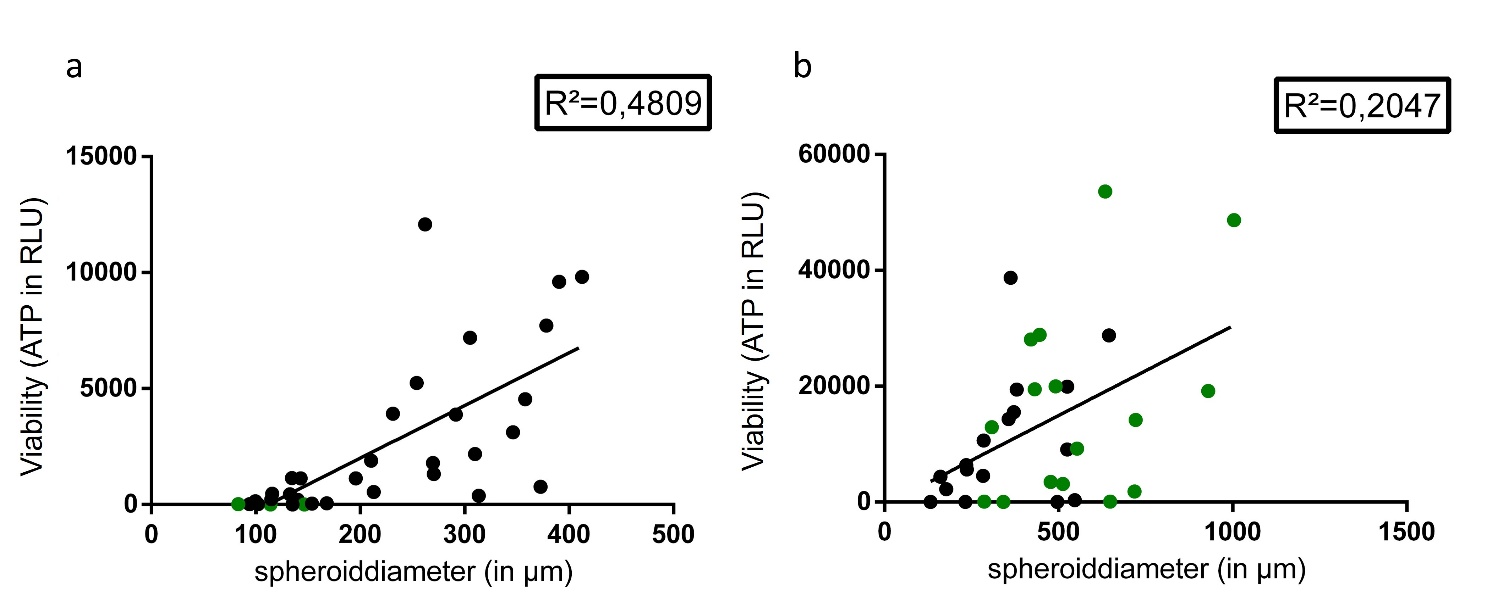


**Supplemental Figure 10 Therapy response of SW480 (a) and SW620 (b) spheroids after incubation with 5-FU for 72 hours.** The Graph shows the viability of treated spheroids after 72 hours of incubation with 5-FU and their diameter before therapy. Spheroid clustering is indicated by colour (loose – green, compact – black).
